# A Comprehensive Evaluation of Multiple Enlarged Perivascular Space Segmentation Tools

**DOI:** 10.1002/alz70856_106612

**Published:** 2026-01-08

**Authors:** James D LeFevre, Dandan Liu, W Hudson Robb, T. Bryan Jackson, Yukti Vyas, Kimberly R. Pechman, Niranjana Shashikumar, Bennett A. Landman, L. Taylor Davis, Timothy J. Hohman, Angela L. Jefferson

**Affiliations:** ^1^ Vanderbilt Memory and Alzheimer's Center, Vanderbilt University School of Medicine, Nashville, TN, USA; ^2^ Vanderbilt University School of Medicine, Nashville, TN, USA; ^3^ Department of Biostatistics, Vanderbilt University Medical Center, Nashville, TN, USA; ^4^ Vanderbilt Memory and Alzheimer's Center, Vanderbilt University School of Medicine, Nashville, TN, USA; ^5^ Department of Electrical and Computer Engineering, Vanderbilt University, Nashville, TN, USA; ^6^ Department of Radiology, Vanderbilt University Medical Center, Nashville, TN, USA; ^7^ Department of Neurology, Vanderbilt University Medical Center, Nashville, TN, USA; ^8^ Department of Medicine, Vanderbilt University Medical Center, Nashville, TN, USA

## Abstract

**Background:**

Enlarged perivascular spaces (ePVS) are a marker of cerebral small vessel disease (SVD) detectable on magnetic resonance imaging (MRI). Due to their small size, diffuse distribution, and similar appearance to other markers of SVD, ePVS are inherently difficult to quantify on MRI, resulting in a lack of reliable quantification methods. We comprehensively evaluated and tested several automated ePVS segmentation algorithms by comparing their detection and segmentation performances to gold standard manual tracings.

**Method:**

Vanderbilt Memory and Aging Project participants (*n* = 30, 72.2±8.8 years, 47% female) underwent T1‐weighted, T2‐weighted, and fluid‐attenuated inversion recovery 3T MRI, and were manually traced by a neuroradiologist to measure ePVS burden. Five algorithms were used to segment ePVS on all participants: BigrBrain, SHIVA‐T1, MAPS‐T1, SegCSVD, and a locally developed nnU‐Net, Detection and Output of enlaRged pErivascular Spaces (DORES). To assess algorithm detection and segmentation capabilities, the Dice score, F1 score, absolute volume difference (AVD), and absolute element difference (AED) were calculated and compared against the expert manual rater separately for the basal ganglia and white matter.

**Result:**

The algorithm with the highest overall performance was DORES, followed by SegCSVD. In the basal ganglia, the DORES achieved a Dice score of 0.73±0.09, F1 score of 0.72±0.10, AVD of 88±120, and AED of 29±33. SegCSVD, by contrast, achieved a Dice score of 0.56±0.09, F1 score of 0.52±0.08, AVD of 109±58, and AED of 52±53. In the white matter, DORES achieved a Dice score of 0.63±0.17, F1 score of 0.68±0.16, AVD of 327±629, and AED of 68±79. By comparison, SegCSVD achieved a Dice score of 0.39±0.18, an F1 score of 0.39±0.18, an AVD of 380±643, and an AED of 135±127 in the white matter.

**Conclusion:**

We present a new deep‐learning algorithm, DORES, as an effective tool for segmenting ePVS in our cohort. Compared to other algorithms, DORES demonstrated higher accuracy, agreement, and validity across metrics of detection, segmentation, and correlations with manual tracings in both basal ganglia and white matter.

